# The Trauma of Peer Abuse: Effects of Relational Peer Victimization and Social Anxiety Disorder on Physiological and Affective Reactions to Social Exclusion

**DOI:** 10.3389/fpsyt.2014.00026

**Published:** 2014-03-18

**Authors:** Benjamin Iffland, Lisa Margareta Sansen, Claudia Catani, Frank Neuner

**Affiliations:** ^1^Department of Psychology, Bielefeld University, Bielefeld, Germany; ^2^Christoph-Dornier-Stiftung für Klinische Psychologie, Bielefeld, Germany

**Keywords:** cyberball, social anxiety disorder, peer victimization, social exclusion, autonomic arousal, physiological indices

## Abstract

**Background:** Social exclusion elicits emotional distress, negative mood, and physiological stress. Recent studies showed that these effects were more intense and persisting in socially anxious subjects. The present study examined whether the abnormal reactions of socially anxious subjects can be traced back to previous experiences of relational peer victimization during childhood and adolescence.

**Methods:** Participants (*N* = 74) were patients with a diagnosis of social anxiety disorder as well as healthy controls. The patient and control groups were subdivided into two subgroups according to the subject’s reports about previous relational peer victimization. Immediate and delayed physiological (skin conductance level and heart rate) and affective reactions to a simulated social exclusion in a ball-toss game (Cyberball) were recorded.

**Results:** Overall, subjects’ immediate reactions to social exclusion were an increase in skin conductance and a reduction of positive affect. Regardless of the diagnostic status, subjects with a history of relational peer victimization showed a more intense self-reported affective change that was accompanied by a blunted skin conductance response. However, the mood of the subjects with a history of peer victimization recovered during a 15 min waiting period. A diagnosis of social anxiety disorder did not affect the reactions to social exclusion on any measure.

**Conclusion:** Findings indicate that stress reactions to social exclusion depend more on previous experiences of peer victimization than on a diagnosis of social anxiety disorder. The findings indicate that memories of negative social experiences can determine the initial stress reaction to social threats.

## Introduction

Aversive experiences during childhood and adolescence can have lasting consequences and contribute to different psychological disorders including depression and anxiety disorders (1–5). Second to traumatic events that involve a threat to life and limb, it is aversive social experiences that can be detrimental for mental health. Traumatic social experiences include emotional abuse and neglect by caretakers (6, 7) as well as non-physical forms of abuse by peers (also referred to as relational peer victimization). Relational peer victimization involves abusive acts like bullying, verbal threats or aggression, malicious manipulation of a relationship, friendship withdrawal, and damaging another’s peer relationships (8). Peer victimization increases the risk of various forms of psychopathology (9) but is particularly linked to sub-clinical social anxiety (8, 10–12) as well as social anxiety disorder (SAD; 13). SAD is a common anxiety disorder that is characterized by fear of social situations and negative evaluation, and caused by an interplay of a genetic vulnerability and negative social learning experiences (14–16).

However, the mechanisms that link experiences of victimization and later psychopathology are still unclear. Recently, models of cognitive vulnerability (17, 18) that are related to memory models of traumatic events (19) as well as network models of emotions (20) have suggested that negative learning experiences may form associative memory representations that may trigger stereotype and pathological reactions in similar social situations. In this understanding, the cognitive vulnerability for social anxiety (17, 18, 21) caused by peer victimization may be modeled as a strong associative memory representation that consists of a network of characteristic stimulus and response elements of the traumatic social situation. Later on, specific environmental features that remind the individual of experiences of relational peer victimization may automatically trigger the recurrence of symptoms of anxiety and fear, even if the current situation is not threatening (18, 22). As a consequence, several social situations are likely to elicit a spectrum of behavioral, autonomic, and endocrine responses that normally only occur in the context of danger (23). In accordance with this idea, Hackmann et al. (24) reported that the majority of patients with social anxiety disorder could identify a particular memory, which they felt was closely linked to the recurrence of symptoms of social anxiety disorder.

This conceptualization predicts that everyday social challenges may provoke more intense reactions in subjects with a history of victimization as such events could trigger associative response elements of the social traumatic experience. With this study we aimed to test this hypothesis. According to Lang (20), the response elements of such associative networks include physiological elements. As a consequence, the responses to social situations that remind the individual of similar earlier experiences should be accompanied by more intense physiological responses in subjects with a history of victimization. While prior studies have shown that subjects with PTSD presented with increased heart rate, skin conductance level (SCL), and blood pressure after presentation of trauma relevant stimuli (25–30), there is currently minimal evidence that these findings can be transferred to non-physical forms of violation, and disorders other than PTSD.

A key element of relational peer victimization is social exclusion (31, 32), which can be experimentally simulated (33–36). For the purpose of this study, we referred to the Cyberball paradigm as an experimental intervention that might serve as a reminder of previous relational victimization. Cyberball is a virtual ball-tossing game (35, 37) where participants are told that they would be playing with two or more co-players on the computer and to mentally visualize the situation, themselves and the other players. In general, the exclusion evokes negative mood and brings about reduced feelings of belonging, self-esteem, control, and meaningful existence, with socially excluded subjects showing a more intense reaction (e.g., 33, 35, 36).

In socially anxious subjects, the Cyberball game elicited immediate and delayed effects on psychological outcomes (38, 39). Highly anxious subjects reported more psychological distress than low anxious participants immediately after the Cyberball game. Moreover, 45 min after the Cyberball game, highly socially anxious subjects still reported more distress than low anxious subjects who had returned to their baseline levels. Additionally, exclusion during Cyberball has been demonstrated to elicit self-regulatory deficits that persisted only in highly anxious subjects forty-five minutes after the exclusion episode (40).

In addition, neuro-imaging studies revealed that processing of social exclusion activates a neural network that is associated to the processing of physical pain (41–43). However, so far there is little research on the peripher-physiological responses to social exclusion. Kelly et al. (44) showed that independent of condition, arousal as measured by SCL decreased over the course of time. However, arousal of the excluded subjects did not decrease to the same extent as the included subjects’ arousal. Consistent with this finding, social exclusion implemented by Cyberball was associated with significant changes in SCL in a sample of 119 female children and adolescents from a private residential summer camp (45). During the Cyberball game, subjects showed heightened SCLs. However, there were no effects of social exclusion on heart rate. The Cyberball game did not elicit physiological reactions as measured by heart rate and cortisol level in a sample of 18 healthy students (46).

The aim of this study was to explore the impact of experiences of peer victimization and social anxiety on physiological and psychological reactions to social exclusion. We postulated that immediately after the Cyberball game subjects reporting a history of relational peer victimization would show higher levels of physiological activation as measured by SCL and heart rate than subjects without peer victimization experiences. Furthermore, subjects high on peer victimization should report less positive affect and more negative affect in reaction to social exclusion. We postulated that these reactions would be stable over a waiting period of 15 min. Furthermore, assuming memory representations are potential mediators between victimizations and pathology, we hypothesized that the effect of a history of victimization would be even stronger for subjects with SAD.

## Materials and Methods

### Participants

Participants were recruited through the Outpatient Psychotherapy Clinic of Bielefeld University and bulletins at the campus of Bielefeld University. Participants included 37 (29 females) individuals who met DSM-IV-TR (47) criteria for a diagnosis of social anxiety disorder and 37 healthy controls (30 females). Exclusion criteria included (a) any current DSM-IV Axis I psychiatric disorder other than social anxiety disorder without a co-morbid SAD, (b) evidence of a current substance abuse or dependence, (c) evidence of current or past psychosis, (d) evidence of acute suicide intention or ideation, and (e) current medication altering physiological reactivity. Moreover, assuming that they might be too suspicious of the experimental manipulation, subjects currently enrolled at the faculty of psychology at Bielefeld University were rejected from participation. The demographic characteristics of the sample are presented in Table 1.

**Table 1:** **Subject characteristics and mean values on the assessments (*N* = 74)**.

	Total (*N* = 74)	Social anxiety disorder + high peer victimization (*n* = 21)	Social anxiety disorder + low peer victimization (*n* = 16)	Healthy controls + high peer victimization (*n* = 16)	Healthy controls + low peer victimization (*n* = 21)	*p*
Age, *M* (SD, range)	24.41 (4.34, 18–37)	25.14 (4.61, 19–36)	25.00 (4.24, 20– 34)	24.94 (4.54, 19– 37)	22.81 (3.84, 18– 33)	N.S.
Gender, % female (*n*)	79.7 (59)	66.7 (14)	93.8 (15)	68.8 (11)	90.5 (19)	N.S.^a^
Family status, % single (*n*)	58.1 (43)	57.1 (12)	62.5 (10)	56.3 (9)	57.1 (12)	N.S.^a^
Educational level, % graduation and higher (*N*)	93.2 (69)	90.5 (19)	93.8 (15)	93.8 (15)	95.2 (20)	N.S.^a^
Employment, % unemployed (*N*)	4.1 (3)	9.5 (2)	6.3 (1)	0.0 (0)	0.0 (0)	N.S.^a^
Co-morbid Axis I psychiatric disorder, % yes (*N)*	12.1 (9)	28.6 (6)	18.8 (3)	0.0 (0)	0.0 (0)	0.011^a^
Psychotherapeutic treatment,% lifetime (*N*)	37.8 (28)	61.9 (13)	50.0 (8)	31.3 (5)	9.5 (2)	0.003^a^
Medication, % psychopharmacological treatment (*N*)	9.5 (7)	23.8 (5)	12.5 (2)	0.0 (0)	0.0 (0)	0.028^a^
Social phobia scale, *M* (SD)	23.58 (15.77)	34.81 (14.51)	32.38 (13.93)	16.69 (10.33)	10.90 (8.42)	0.000
Social interaction anxiety scale, *M* (SD)	34.20 (17.57)	48.67 (12.38)	45.38 (13.40)	24.13 (8.88)	18.90 (11.55)	0.000
Peer victimization, *M* (SD)	14.27 (7.70)	22.10 (5.44)	9.13 (2.60)	18.69 (4.25)	7.00 (3.38)	0.000
Childhood trauma questionnaire, *M* (SD)	39.57 (11.49)	44.00 (12.71)	46.13 (12.35)	36.88 (9.42)	32.19 (4.64)	0.000^b^
Emotional abuse, *M* (SD)	9.54 (4.48)	11.24 (5.25)	12.13 (4.90)	8.75 (3.19)	6.48 (1.08)	0.000
Emotional neglect, *M* (SD)	10.92 (4.31)	13.33 (4.09)	13.06 (4.84)	9.25 (3.38)	8.14 (2.20)	0.000
Physical abuse, *M* (SD)	6.35 (2.46)	7.29 (3.29)	6.50 (2.34)	6.25 (2.44)	5.38 (0.86)	N.S.
Sexual abuse, *M* (SD)	5.53 (1.41)	5.24 (0.70)	6.19 (2.43)	5.31 (0.60)	5.48 (1.25)	N.S.
Beck depression inventory, *M* (SD)	11.83 (7.70)	16.15 (9.19)	12.50 (8.63)	12.56 (4.11)	6.40 (3.69)	0.000
Brief symptom inventory – global severity index, *M* (SD)	0.79 (0.53)	1.18 (0.64)	0.84 (0.47)	0.75 (0.35)	0.40 (0.23)	0.000

*^a^Chi-Quadrat-Test*.

*^b^CTQ sum score for the 28-item version*.

### Instruments

#### Diagnosis of social anxiety disorder

Diagnostic status was assessed using the German Version of the Mini International Neuropsychiatric Interview (MINI; 48–50). The MINI is a structured clinical interview designed to generate diagnoses for the main diagnostic and statistical manual (DSM)-III-R/IV Axis I disorders. The interviews were conducted by Master-level clinical psychologists who were trained in the application of the MINI.

#### Social anxiety symptoms

For the assessment of the severity of symptoms of social anxiety disorder, the German version of the social phobia scale/social interaction anxiety scale (SPS/SIAS; 51) was used. The SPS was developed to assess anxiety related specifically to social performance, whereas the SIAS was designed to measure anxiety related to general social interaction. Both, the SPS and the SIAS consist of 20 items using a five-point Likert scale that are rated from 0 (not at all) to 4 (extremely) indicating how characteristic or true the statements are for the respondent. On both scales total-scores range from 0 to 80. Cut-off scores of 20 on the SPS and 30 on the SIAS indicate a clinically relevant level of social anxiety (52). The German version of the SPS/SIAS has shown high levels of internal consistency and convergent, but deficient discriminant validity (51).

#### Relational peer victimization

To assess relational peer victimization, we used a checklist that was recently developed and validated in our working group (53). The Fragebogen belastender Sozialerfahrungen (FBS; questionnaire on stressful social experiences) assesses various forms of abusive experiences occurring amongst peers. It consists of a list of 22 aversive social situations (i.e., “I was excluded from games or activities by other children or adolescents,” “I have been laughed at in the presence of other children”). For each situation respondents are asked whether or not (Yes or No) they have experienced this situation during childhood (age of 6–12) or adolescence (age of 13–18). The total-score is calculated as a sum of “Yes” responses in of both age periods and ranges from 0 to 44. The total-score showed a satisfying stability over a period of 20 months (*r* = 0.89). Construct validity has been confirmed through correlations with measures of psychological symptom distress and social anxiety. Moderate correlations with the scales of the CTQ and an incremental contribution to the prediction of psychopathology indicate that the FBS assesses an additional construct of child maltreatment.

#### Child maltreatment

The German version of the childhood trauma questionnaire (CTQ; 54) was used to assess different types of childhood maltreatment. The 28-item self-rated scale distinguishes five areas of maltreatment (sexual abuse, emotional neglect, emotional abuse, physical neglect, and physical abuse). The items are rated from 1 (never true) to 5 (very often true) with a possible range of subscale scores of 5–25. The psychometric properties of the German version were similar to the original version and it has been shown to be a reliable and valid screen for childhood maltreatment. Internal consistency of all scales except physical neglect was shown to be high (Cronbach’s α > 0.89). Correlations with self-reported measures for post-traumatic stress, dissociation, and general psychopathology were low to moderate (54). However, in a recent validation study from the general population (55), the five factor structure of the original version showed only a moderate fit. As the physical neglect subscale was highly correlated with the other subscales, presented with a weak internal consistency in comparison to the other subscales, and since the fit of a four factor structure excluding the physical neglect items was much superior to the five factor model, the physical neglect subscale and its items were disregarded in the following analyses (55). In the present study, Cronbach’s alpha was α = 0.73 for all items. Internal consistency of all scales was acceptable to high (Cronbach’s α > 0.75).

#### Symptoms of depression

Symptoms of depression were measured using the German version of the Beck depression inventory (BDI-II; 56). The self-report measure consists of 21 items relating to symptoms of depression. The items are rated on a four-point scale indicating the severity of symptoms and are considered to be rated for the experience of the past 2 weeks including today. Higher scores indicate more severe depressive symptoms. The BDI-II has shown good psychometric properties in clinical and non-clinical samples (57).

#### General psychopathology

In order to measure psychopathology and psychological distress, the German version of the brief symptom inventory (BSI; 58–60) was used. The BSI is a 53-item short form of the symptom check List 90 (SCL-90). It produces the same nine primary symptom dimensions (somatization, obsessive-compulsivity, interpersonal sensitivity, depression, anxiety, hostility, phobic anxiety, paranoid ideation, and psychoticism). Furthermore, three global indices measure general psychological distress. These include the global severity index (GSI), the positive symptom total (PST), and the positive symptom distress index (PSDI). Each item is rated on a five-point Likert scale ranging from 0 (not at all) to 4 (extremely) and is considered to be rated for the experience of the past 7 days including today.

#### Positive and negative affect

For the assessment of positive and negative affect in reaction to social exclusion, the German version of the positive and negative affect schedule was used (PANAS; 61, 62). The PANAS was developed to assess positive and negative affect measured on a five-point Likert scale ranging from 1 (very slightly) to 5 (extremely). It is intended to gain information on a participant’s emotional state at the moment that the questionnaire is given. The positive and negative affect scales consist of 10 items each. The scales were shown to be largely uncorrelated (62). The German version showed good internal consistency (Cronbach’s α > 0.84; 61).

#### Manipulation checks

There were two manipulation checks to confirm participants’ perception of their inclusionary status. First, they were asked to estimate the percent of throws they had received (“Assuming that 33% of the time you would receive the ball if everyone received it equally, what percent of the throws did you receive?”). Second, they were asked to rate how much they felt excluded while playing the Cyberball game on a nine-point Likert scale ranging from 1 (very included) to 9 (very excluded). In addition, participants had the chance to write down their thoughts during the Cyberball game (35) and to comment on their thoughts.

#### Procedure

The study was conducted within two sessions. The second session was scheduled on average 10 days after the first session. At the first session, participants provided written informed consent at the beginning of the assessment. The consent form stated that the purpose of the study was to “evaluate the relationship of mental visualization and psychological distress.” Subjects were informed that participation was voluntary, and that they could discontinue at any time. Following, participants were administered the MINI (49) to determine diagnostic status. Afterward, they were invited to fill in a socio-demographic questionnaire as well as the study questionnaires (see below). At the second session, skin conductance and electrocardiogram (ECG) leads were positioned on participants with the assistance of the research assistants. To assess skin conductance, 9 mm electrodes were attached to the thenar and hypothenar surface of the non-dominant hand. A layer of an isotonic electrolyte gel was placed on the electrodes to increase conduction. For the assessment of heart rate signals, participants placed three disposable Ag/AgCl electrodes on the manubrium sterni, the lowest part of the sternum and the lowest left rib. Skin conductance and heart rate were registered and digitized using a Varioport biosignal recording device (Becker Meditec, Karlsruhe, Germany), that was controlled by a Windows computer with Variograph software (Becker Meditec, Karlsuhe, Germany). Skin conductance and heart rate were recorded simultaneously with a sampling rate of 512 Hz. Skin conductance signal was converted to microsiemens (μS) and heart rate signal to beats per minute (bpm). Skin conductance was missing for three participants and heart rate for four due to experimenter error.

Baseline physiological activity (skin conductance and heart rate) was assessed during a 3-min period of rest. During this period, participants were instructed to sit quietly and relax. Afterwards, participants were asked to fill in baseline assessments of affect. Next, participants were informed that to practice and test mental visualization, they would be playing a virtual ball-tossing game called “Cyberball” with what they believed to be two other players (35). In reality, these players were computer generated. Participants were instructed to mentally visualize (as vividly as possible) the scene throughout the game (“Imagine what the others look like. What sort of people are they? Where are you playing? Is it warm and sunny or cold and rainy?”). Shortly after the instruction, the experimenter received a staged phone call informing them that the other players were ready to start. Then the game began.

At the beginning of the game, the participants received the ball and were then required to indicate to whom they would like to throw the ball by clicking on the appropriate player icon. After receiving the ball twice, participants were excluded from the game and did not receive the ball ever again. The game lasted for a total of 30 throws. Following the game, participants filled in the affect scales and the manipulation checks (see above).

After completing the questionnaires, participants were told that the experimenter would have to check recordings of the physiological signals of the other players and were instructed to stay on their chair and wait until the experimenter returned to remove the electrodes. At the end of a waiting period of 15 min, the experimenter returned and asked the participants to fill in the affect scales. Skin conductance and heart rate were recorded continuously throughout the ball-tossing game and the waiting period.

Finally, participants were debriefed and had the chance to comment on the study and ask questions of the researchers. The study was approved by the Ethical Committee of the Department of Psychology of Bielefeld University.

#### Data reduction and analyses

Physiological data were pre-processed and analyzed using MATLAB version 7.7 (2008b, The MathWorks, Natick, MA, USA) with the toolboxes ANSLAB (63) and Ledalab (available under www.ledalab.de). R-waves in the ECG data were identified automatically by ANSLAB software (63) and converted to bpm. Additionally, a visually artifact inspection was conducted. Artifactual data points were manually replaced, non-recognized R-waves were edited and sections with high proportions of artifacts were not evaluated. Similarly, raw data of skin conductance were screened for implausible artifacts and manually edited. For further analyses, mean levels of skin conductance and heart rate, respectively, during baseline (3 min), the Cyberball game (3 to 5 min) and the waiting period (15 min) were used.

#### Statistical analyses

All statistical analyses were carried out using the Statistical Package for the Social Sciences SPSS 20. To test the reactions to social exclusion dependent on the extent of social anxiety and peer victimization, 2 (social anxiety disorder vs. control group) × 2 (high vs. low peer victimization) × 2 (time: baseline, Cyberball and Cyberball, waiting period) analyses of variance (ANOVAs) with repeated measurement on the third factor were conducted. In a first step, effects of social exclusion were analyzed regarding assessments at baseline and during/after the Cyberball game. In a second step, assessments during/after the Cyberball game and during/after the waiting period were used to analyze effects of the waiting period. Additionally, all ANOVAs were carried out as analyses of covariance (ANCOVAs) with the CTQ sum score serving as covariate to control for the influence of childhood maltreatment. Similarly, to control for the influence of co-morbid psychiatric disorders on the reactions to social exclusion ANCOVAs with the diagnostical status of a current major depressive disorder (MDD) serving as covariate were carried out. As the pattern of results did not change, ANOVAs are reported.

## Results

The total sample consisted of 74 subjects, predominantly women (*n* = 59, 79.7%), of which 37 individuals (50.0%) were diagnosed with social anxiety disorder. In nine individuals diagnosed with social anxiety disorder a co-morbid current MDD was determined. The average age was *M* = 24.41 (SD = 4.34). Table 1 presents participants’ means on the assessments.

### Skin conductance level

**Figure 1:**
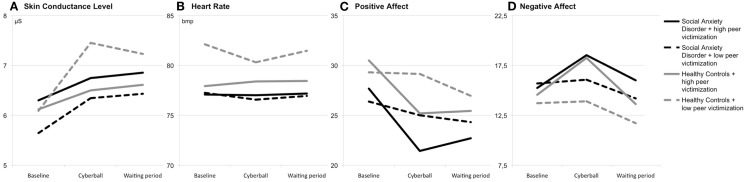
**Group means of skin conductance level (A), heart rate (B), positive affect (C), and negative affect (D) at baseline, during/after the Cyberball game and during/after the waiting period**.

For SCL reactivity to social exclusion, the ANOVA showed a significant interaction of time and peer victimization, *F*(1, 68) = 4.83; *p* = 0.031; η^2^ = 0.066. The interaction of time and social anxiety did not reach significance, *F*(1, 68) = 1.12; *p* = 0.293; η^2^ = 0.016. In addition, the ANOVA showed a significant main effect of time, *F*(1, 68) = 26.42; *p* < 0.001; η^2^ = 0.280. For the waiting period, neither the interaction of time and peer victimization, *F*(1, 68) = 1.01; *p* = 0.318; η^2^ = 0.015, nor the interaction of time and social anxiety, *F*(1, 68) = 0.77; *p* = 0.384; η^2^ = 0.011, were significant. Furthermore, there was no significant main effect of time, *F*(1, 68) = 0.06; *p* = 0.809; η^2^ = 0.001 (see Figure 1A).

### Heart rate reactivity

For heart rate reactivity to social exclusion, the ANOVA showed no significant effects [time: *F*(1, 66) = 0.40; *p* = 0.531; η^2^ = 0.006; time × peer victimization: *F*(1, 66) = 0.94; *p* = 0.336; η^2^ = 0.014; time × social anxiety: *F*(1, 66) < 0.01; *p* = 0.977; η^2^ < 0.001]. Similarly, no significant effects were found for the waiting period [time: *F*(1, 65) = 1.21; *p* = 0.275; η^2^ = 0.018; time × peer victimization: *F*(1, 65) = 0.56; *p* = 0.459; η^2^ = 0.008; time × social anxiety: *F*(1, 65) = 0.12; *p* = 0.728; η^2^ = 0.002] (see Figure 1B).

### Positive affect

On the ratings of the positive affect, the ANOVA showed a significant interaction of time × peer victimization, *F*(1, 70) = 11.75; *p* = 0.001; η^2^ = 0.144. There was no significant interaction of time × social anxiety, *F*(1, 70) = 0.53; *p* = 0.471; η^2^ = 0.007. In addition, the ANOVA revealed a significant main effect of time, *F*(1, 70) = 20.28; *p* < 0.001; η^2^ = 0.225. No significant interaction of time × peer victimization, *F*(1, 70) = 3.76; *p* = 0.057; η^2^ = 0.051, was found in the ANOVA for the waiting period. Furthermore, the ANOVA showed no significant interaction of time × social anxiety, *F*(1, 70) = 1.24; *p* = 0.269; η^2^ = 0.017, and no significant main effect of time, *F*(1, 70) = 0.35; *p* = 0.557; η^2^ = 0.005 (see Figure 1C).

### Negative affect

The ANOVA showed a significant interaction of time × peer victimization, *F*(1, 70) = 6.07; *p* = 0.016; η^2^ = 0.080, for the ratings of negative affect. No significant effect was found for the interaction of time x social anxiety, *F*(1, 70) = 0.01; *p* = 0.934; η^2^ < 0.001. However, the ANOVA showed a significant main effect of time, *F*(1, 70) = 8.41; *p* = 0.005; η^2^ = 0.107. For the waiting period, neither the interaction of time × peer victimization, *F*(1, 70) = 1.71; *p* = 0.195; η^2^ = 0.024, nor the interaction of time x social anxiety, *F*(1, 70) = 1.05; *p* = 0.309; η^2^ = 0.015, were found to be significant. However, the ANOVA showed a significant main effect of time, *F*(1, 70) = 22.63; *p* < 0.001; η^2^ = 0.244 (see Figure 1D).

## Discussion

In a sample of subjects diagnosed with social anxiety disorder and healthy controls, we found that reactions to an episode of social exclusion were primarily influenced by the degree of relational peer victimization rather than by the diagnosis of SAD. While an increase in skin conductance immediately after the exclusion was observed for all groups of subjects, this physiological response was attenuated among the subjects with a history of peer victimization, while the affective responses were more intense. However, after a waiting period of 15 min the positive affect of the group of subjects with victimization increased again.

In general, social exclusion causes a physiological stress response. Across all subjects, we observed an increase in skin conductance immediately after the exclusion simulation. As our study lacks a control group of non-excluded subjects, we cannot exclude that the increase of skin conductance at this time point can be attributed to confounding factors. However, higher SCLs were reported in the exclusion than in the inclusion condition (44). Kelly et al. (44) proposed that these higher arousal levels are linked to stress associated with social pain, arguing that brain regions associated with increases in SCLs [i.e., anterior cingulated cortex (64)] are also activated when subjects were experiencing social exclusion (42). Moreover, the same regions are activated when individuals were confronted with the distress of physical pain (65) and loss of social connections (see 66 for review). However, the physiological response to social exclusion was restricted to skin conduction, we could not observe any effect on heart rate. In accordance with this, Krimsky (46) reported no differences in heart rate variability and cortisol level between included and excluded subjects after the Cyberball game. Krimsky (46) suggested that the Cyberball paradigm was not effectively powerful in eliciting substantial and consistent physiological responses.

The immediate affective and physiological reaction to social exclusion is predicted by previous experiences of relational peer victimization. While we confirmed the hypothesis that subjects with a history of victimization present with a more intense affective reaction, their physiological response was, contrary to our expectations, attenuated rather than increased. This finding indicates that repetitive experiences of peer victimization may produce a qualitative rather than quantitative change of the autonomic nervous system response to social challenges. This idea is consistent with a large number of previous studies finding that the experience of adverse life events alters the regulation of the neurohormonal stress response (67–69). Healthy subjects with a history of childhood maltreatment showed blunted cortisol responses to psychological stress and diminished reactions to direct endocrine challenges (70–77). Similar patterns of cortisol responses to stressors were found for relational peer victimization (78, 79). In addition, Lovallo et al. (80) studied the impact of maltreatment on the autonomic nervous system and found that subjects who experienced adverse life events in childhood and adolescence showed not only an attenuated cortisol but also a blunted heart rate response. Recent findings indicated blunted rather than increased physiological responses in patients with multiple-trauma PTSD (81–83). Taken together, there is reason to conclude that repeated adversities including peer victimization cause a dissociation of the affective and physiological response to social stressors, with an intense self-reported affective change accompanied by a blunted physiological reaction. So far, it is unclear whether this reduction of physiological response is caused by a single mechanism that controls the different stress axes (84, 85), or whether a simultaneous change of the functioning of the single axes themselves, as currently discussed for the hormonal HPA axis (67–69) is responsible for this effect. However, the qualitative difference of the responses shows that it is too simplistic to assume a single conditioning or associative mechanism to explain the effects of repeated aversive experiences like peer victimization.

Contrary to our expectations and to previous findings (38–40) we could not find any influence of social anxiety on the responses to social exclusion. This finding contrasts with previous evidence of increased reactions (i.e., enhanced skin conductance, heart rate, and potentiated startle) to social threat in subjects with social anxiety (86–88). However, as peer victimization is highly linked to social anxiety disorder (13) it may be speculated that the association of social anxiety and pathological responses to social exclusion presented in previous studies may be produced by experiences of peer victimization or emotional maltreatment rather than psychopathology per se. In addition, relational peer victimization caused increased sensitivity to social exclusion even when analyses incorporated experiences of child maltreatment, suggesting that experiences of peer victimization have an independent and specific impact on reactions to social exclusion.

The present study has several limitations. Assessment of peer victimization and child maltreatment was based on retrospective accounts and self-report, both of which are subject to recall biases (89). Subjects with social anxiety may be more likely to be biased in estimating the occurrence of negative social situations. However, analyses of the validity of retrospective reports showed that distortions are present but not sufficiently large enough to invalidate retrospective studies (90). Moreover, in retrospective assessments of childhood maltreatment, under-reporting was more prevalent than over-reporting. The present study used levels of skin conductance and heart rate as markers for physiological reactions to social exclusion. Although skin conductance and heart rate reactivity is associated with changes in brain regions regulating the HPA axis, a more direct assessment of HPA axis responses (e.g., cortisol measures) in patients with SAD would be desirable. In addition, our study was limited by the use of the Cyberball paradigm to induce social exclusion. As mentioned above, previous studies reported that the Cyberball paradigm was not sufficient to evoke physiological responses (45, 46). Other paradigms that involve direct communication, negative evaluation, and rejection may be more powerful. One example would be the Trier social stress test (TSST; 91), which has been shown to cause increases in heart rate and cortisol as well as subjective reports of psycho-social stress (92). Unlike prior studies on the long-term effects of social exclusion in socially anxious subjects, the present study used a waiting period of 15 minutes instead of a 45-min-delay. Testing potential additional effects of peer victimization and social anxiety after a longer delay would be a desirable extension of the present study. Finally, generalizability of our findings is restricted by the high rate of female subjects. Although social anxiety disorder is more prevalent among women (93–95), prevalence rates were found to be equal for both sexes in clinical samples. A more appropriate sex ratio would be worthwhile in further investigations.

## Conclusion

Experiences of social exclusion cause immediate affective and physiological reactions. Although the effects of being rejected are not linked to psychopathology or previous social experiences in general, the extent, and continuity of affective and physiological responses is determined by prior experiences of peer victimization rather than social anxiety. In addition, the present study is consistent with previous reports indicating the existence and significance of associative networks and associative memory processing (19, 20), reflecting processes that may be considered as social traumatization. However, non-associative memory mechanisms (96) and epigenetic mechanisms (97, 98) might also contribute to the effects of emotional traumatization.

## Conflict of Interest Statement

The authors declare that the research was conducted in the absence of any commercial or financial relationships that could be construed as a potential conflict of interest.
